# Death from Purulent Infection

**Published:** 1865-09

**Authors:** 


Death from Purulent Infection.—The French journals, says the *Brit. Med. Journal*, announce the death of M. Bauchet, surgeon, of St. Antoine, from a poisoned wound of a finger resulting from dressing a purulent sore of a patient. A writer in *L'Union Medicale* says:

"If he had performed his hospital dressing with chloride of sodium, permanganate of potass, or alcohol, we should probably not have to deplore the death of this young and deserving *confrere.* In 1831, I punctured my finger while dissecting. Notwithstanding caustic and fomentations, I had, two days afterward, shiverings, headache, nausea—all the signs of purulent infection. I went to Breschet for advice; and he said, 'Go at once to the Café Procope, order half a bowl of punch, drink it burning hot, and run home to bed.' I followed the prescription to the letter, and was in fact, helped home by two friends. It was the only time in my life I ever got tipsy. I slept fifteen hours, and during the whole time sweated profusely. On the following day, all the symptoms of septicæmia, as M. Piorry would say, had disappeared."—*Med. and Surg. Reporter.*

